# Global, regional and national estimates of coexisting forms of malnutrition among the neonates, infants and young children – A secondary data analysis of Demographic & Health Surveys (DHS) from 2006 to 2024.

**DOI:** 10.12688/f1000research.172154.1

**Published:** 2025-12-19

**Authors:** Asif Khaliq, Bushra Ashar, Haji Abdul Rehman, Mohammad Junaid, Yusra Rizwan, Namra Aijaz, Sibgha Fawad Memon, Nazeer Ahmed, Muhammad Hamza, Ubaidullah Khan, Abdul Haseeb, Rida Shakeel, Izzan Ahmed Usmani, Mohammad Javad Davoudabadi

**Affiliations:** 1 Queensland University of Technology School of Public Health and Social Work, Kelvin Grove, Queensland, Australia; 2 Dow University of Health Sciences, Karachi, Sindh, Pakistan; 3 Karachi Medical and Dental College, Karachi, Sindh, Pakistan; 4 CMH Multan Institute of Medical Sciences, Multan, Punjab, Pakistan; 5 Khyber Medical College, Peshawar, Khyber Pakhtunkhwa, Pakistan; 6 Ziauddin University Faculty of Medicine, Karachi, Sindh, Pakistan; 7 FMH College of Medicine and Dentistry, Lahore, Punjab, Pakistan; 8 Peoples University of Medical and Health Sciences for Women, Nawabshah, Sindh, Pakistan; 9 University of Health Sciences Lahore, Lahore, Punjab, Pakistan; 10 Queensland University of Technology School of Mathematical Sciences, Brisbane, Queensland, Australia

**Keywords:** Coexisting forms, Malnutrition, Neonate, Infants, Children, Prevalence, Determinants.

## Abstract

**Objectives:**

To estimate Global, Regional, and National prevalence of Malnutrition, specifically Coexisting forms of malnutrition (CFM) and its various types: Coexistence of underweight with stunting (CUS), Coexistence of underweight with wasting (CUW), Coexistence of underweight with wasting & stunting (CUWS), Coexistence of stunting with overweight/obesity (CSO) among children under five years in low- and middle-income countries (LMICs).

**Methods:**

This cross-sectional observational study utilized latest nationally representative Demographic and Health Surveys (DHS) datasets of 62 LMICs corresponding to six global regions from 2006 to 2024. Data of 541,707 children aged 0 to 59 months with complete anthropometry were analysed descriptively (prevalence estimates) and inferentially (multivariate logistic regression).

**Results:**

The global malnutrition prevalence among children was 43.9%, of which CFM was observed among 20.9% children. Among various CFM types, CUS was highly prevalent (11.8%), followed by CUW (4.2%), CUWS (3.2%), and CSO (2.1%). South & Southeast Asian had highest CFM prevalence of 29.6%, while Central Asia and Latin America & Caribbean reported the lowest CFM prevalence of 8.1% and 6.2%, respectively. Among all the LMIC included, Timor-Leste exhibited the highest CFM at 41.8%. In general, children aged between 12 to 35 months had 2-3 times higher odds of various forms of CFM. However, female sex, maternal education, improvement in socioeconomic status, medium to large family size showed significantly lower odds with various forms of CFM.

**Conclusions:**

This study advances the understanding of CFM’s prevalence, determinants, and regional variations, filling a critical gap in global nutrition research. The findings serve as a reminder to reinvest in efforts to protect children from malnutrition during their early years of life. Governments and other global health sector must invest in a well-established system of nutrition surveillance for addressing CFM, informing targeted interventions to improve child health outcomes in LMICs.

## What is already known on this topic

Infants and young children nutrition assessment has historically been a target of various national and international development agendas. Children below five years, particularly those living in low-income and middle-income countries (LMICs) are susceptible to various forms of nutritional disorders. Anthropometry is the most simple, cost-effective and non-invasive nutrition assessment tool, which is often used to assess the nutritional status of children at national and regional level. Nevertheless, despite its widespread implementation, most of the local, national, and international surveys and reports have predominantly documented standalone forms of malnutrition (wasting, stunting, underweight, overweight/obesity). In contrast, the prevalence and determinants of the Coexisting forms of malnutrition (CFM) remain unexplored.

## What this study adds

This is the first study, which measured the national, regional and global prevalence of CFM and its various types among neonates, infants and young children of 62 LMICs using the Demographic & Health Surveys (DHS) datasets. The findings reveal significant inequalities among different regions, with South Asia & Southeast Asia had highest CFM prevalence, where the nutritional adversities were homogenous among almost all countries. In contrast, a glaring nutritional status gap was observed within countries of Latin America & Caribbean region, Sub-Saharan Africa region, and North Asia, West Africa and Europe region. Children aged 12 to 35 months had two-to-three-fold higher risk of CFM than young children – highlighting the need for targeted interventions focusing on feeding practices, micronutrient supplementations, food fortification, and food subsidiaries.

## How this study might affect research, practice or policy

This study proposed a standard for measuring CFM among the neonates, infants, and young children aged below five years using existing anthropometric indices, such as length/height-for-age (LAZ/HAZ), weight-for-age (WAZ), and weight-for-length/height (WLZ/WHZ). The findings of this study underscored the importance of national nutritional estimates and its various types, including CFM, using existing national and regional datasets. Furthermore, this study paves the way to explore sub-clinical cases of CFM among children under five, offering stakeholders (program managers, policy makers, clinicians and global health researchers) an opportunity to revise the existing nutrition specific interventions to combat CFM among young children.

## Introduction

Malnutrition in children is an integral obstacle for achieving the optimal health and nutritional indicators. ^1, 2^ Children under five years are particularly vulnerable to malnutrition due to their rapid growth and increased nutritional needs. ^3^ Stunting, wasting, underweight, and overweight/obesity manifests the common types of malnutrition, which can be assessed by simple and cost-effective anthropometric measurement. ^4^ In children, malnutrition increases the risk of infection and neurodevelopmental impairment, leading to reduced economic productivity during adulthood. ^4– 6^

Worldwide, more than 230 million children below five are malnourished. ^7, 8^ The problem is particularly emerging in low-and-middle-income countries (LMICs), driven by food insecurity, poverty, illiteracy, rural residence, limited healthcare access, and high burden of communicable diseases. ^4, 9– 11^ South Asian (SA) and Sub-Saharan African (SSA) countries carry greatest burden of malnutrition, ^12^ where more than half of children below five years are malnourished. ^13, 14^

Malnutrition in children may exist either standalone or in conjunction with other forms. Children experiencing two or more forms of nutritional problems simultaneously are classified as having the Coexisting forms of malnutrition (CFM). ^4^ Different international and independent bodies underscored CFM as a pressing public health concern. ^4, 15^ Worldwide, all the national and international surveys presented each form of malnutrition separately, ^15– 17^ but the Global Nutrition Report (GNR) in 2014 introduced concept of CFM among stunted children. ^4, 18^ This concept of GNR was partially carried forward by Ferreira (2020), Fongar, et al., (2019), and Varghese, et al. (2019), i.e., they all examined coexistence of stunting with overweight/obesity. ^19– 21^ Since 2020, the World Health Organization (WHO) raised concern of CFM among underweight children, i.e., an underweight child is at risk of either stunting or wasting or both. ^7^ Still, the CFM remains an unexplored or least priority area to address child malnutrition. ^4^

CFM in children is associated with higher mortality compared with children with standalone forms of malnutrition (SFM), i.e., the risk of death in children with SFM is 4 to 8 times higher than a well-nourished child, which increased to over 10 folds in children with CFM. ^22– 24^ Most of the previous studies examined CFM either on a particular country or in a small range of ages. ^4, 15, 23, 24^ Many nutrition surveillance systems, particularly those developed under the Millennium Development Goals (MDGs), have historically focused on single indicators such as stunting, wasting or underweight, but overlooked the critical and complex intersection of CFM in a single child. ^25^ Recognizing this gap, the Sustainable Development Goals (SDGs) highlighted the imperative to “end all forms of malnutrition” by 2030 (SDG 2.2), thereby emphasizing the need for integrated and multi-sectoral action to tackle the multiple dimensions of this problem. ^26^

Given the urgency in addressing this important issue and the necessity for actionable, high-quality data, this study sought to systematically provide up-to-date global, regional, and national estimates of CFM in children-under-five using DHS data from 62 LMICs covering period from 2006 to 2024. Our study aims to provide a guide to policymakers and practitioners to develop effective and equity-focused interventions. The goal remains crystal clear: to make sure that no child is left behind in the broader global effort to accomplish the SDGs and eliminate all forms of malnutrition in all its ramifications.

## Methods

### Study design and data source

In this study, secondary data analysis of nationally representative Demographic and Health Surveys (DHS) conducted between 2006 and 2024 across 62 LMICs was carried out. The DHS program employs standardized methodologies across countries, allowing for harmonized estimates at the national and regional levels. ^27^

### Study datasets, study population and their eligibility criteria

The United States Agency for International Development (USAID) in collaboration with national agencies of LMICs has implemented DHS to around 90 LMICs. ^15^ In this study, data of only those LMICs was considered, which contained data on paediatric anthropometry collected on or after the year 2006. The year 2006 was taken as a reference, because the recent growth standards recommended by the WHO were released in this year. ^28^

Considering the eligibility criteria of this study, a total of 62 DHS datasets were included in the analysis, representing six global regions categorized according to WHO and World Bank classifications: South Asia & Southeast Asia (SASEA), Sub-Saharan Africa (SSA), Latin America & the Caribbean (LAC), North Africa, West Asia & Europe (NAWAE), Central Asia (CA), and Oceania. To maintain consistency and ensure the most up-to-date estimates, only the most recent DHS survey from each country was selected for inclusion. A comprehensive list of countries and survey years is provided in ***Supplementary file-1.*** ^29^

From each included dataset, data of children aged between 0 to 59 months with complete and biologically plausible anthropometric measurements were used for analysis. However, anthropometric outliers were excluded from the analysis, as defined by the WHO growth standards based on the z-score values: ±6.00 S.D. for length/height-for-age z-score [LAZ/HAZ]; ± 5.00 S.D. for weight-for-length/height z-score [WLZ/WHZ]; while for weight-for-age z-score [WAZ], the z-score value of less than −6.00 S.D. or greater than +5.00 S.D. ^30^

### Measurement of study outcomes

In this study, CFM in children was the primary outcome, which was operationalized through three anthropometric indices calculated using WHO AnthroCal ^®^ software (version 3.2.2) based on the 2006 WHO growth standards. These included LAZ/HAZ, WAZ, and WLZ/WHZ. Children were categorized as normal, stunted, wasted, underweight, or overweight/obese based on their z-score. For assessing the CFM prevalence in children, computational coding and categorization of each anthropometric index was performed. Thereby producing nine different types of nutritional status: normal; stunting; wasting; underweight; overweight/obese; coexistence of underweight with stunting (CUS); coexistence of underweight with wasting (CUW); coexistence of underweight with both wasting and stunting (CUWS); and coexistence of stunting with overweight/obesity (CSO). ^29^ Further definitions about each type of anthropometric outcome are presented in Table 1.

**Table 1.** Definition, assessment methods and criteria for coexisting forms of malnutrition (CFM) and its various types.

CFM and its types	Definition	Assessment criteria	Assessment scale
CFM	Presence of stunting or wasting or both among underweight children OR Presence of overweight or obesity among stunted children	LAZ/HAZ and WAZ and WLZ/WHZ	Deviation of at least two indicators: LAZ/HAZ, WAZ and WLZ/WHZ from the normal z-score value ranged between -1.99 S.D. and +1.99.
CUW	Simultaneous presence of wasting among underweight children		Value of WAZ and WLZ/WHZ should be less than equal to -2.00 S.D.
CUS	Simultaneous presence of stunting among underweight children		Value of WAZ and HAZ should be less than equal to -2.00 S.D.
CUWS	Simultaneous presence of wasting and stunting both among underweight children		Value of WAZ LAZ/HAZ and WLZ/WHZ should be less equal to than -2.00 S.D.
CSO	Simultaneous presence of either overweight or obesity among stunted children		Value of LAZ/HAZ should be less than equal to-2.00 S. D, while the value of WAZ and/or WLZ/WHZ should be greater than equal to +2.00 S.D.

CFM = coexisting forms of malnutrition, CUW = coexistence of underweight with wasting, CUS= coexistence of underweight with stunting, CUWS = coexistence of underweight with both wasting and stunting, CSO = coexistence of stunting with overweight/obesity, LAZ/HAZ = length for age/height for age z-score, WAZ = Weight for age z-score, and WLZ/WHZ = weight for length/weight for height z-score.

### Study covariates

A comprehensive set of covariates at the child, maternal, and household levels was included to account for potential confounding.

Child-level variables included age in months, sex, birth order, perceived size at birth, and recent illness. Maternal-level variables included age at childbirth, educational attainment, body mass index, employment status, number of antenatal care visits, and media exposure. Household-level covariates included socioeconomic status (SES), residence, geographic region or province, access to improved drinking water and sanitation facilities, household size, and the number of children under five years.

### Data management and data analysis

Initially, data of each country was screened thoroughly. Datasets with completed information about the paediatric population and their anthropometric measurement were included. However, data devoid of child information was excluded. Following data screening, data harmonization was carried out, in which data of all the countries and regions were merged. Furthermore, data coding and data categorization were also performed.

Descriptive statistics were used to summarize demographic and nutritional characteristics. Frequencies and proportions were calculated for categorical variables. Sample weights were applied to estimate the weighted prevalence of each form of malnutrition, including CFM at global, regional, and national levels.

Bivariate associations between covariates and each type of CFM were assessed using Pearson’s chi-square test, with statistical significance set at p ≤ 0.05. Multinomial logistic regression models were then developed to identify factors associated with four coexisting malnutrition types: CUS, CUW, CUWS, and CSO. Underweight children were used as the reference group in models addressing overlapping undernutrition (CUS, CUW, & CUWS) and stunted children were used as the reference in models addressing CSO. Both unadjusted odds ratios (ORs) and adjusted odds ratios (AORs) with 95% confidence intervals (CI) were estimated. Non-significant covariates were removed from the multivariate model using backward elimination methods until the final model contained only the significant covariates. Multicollinearity was checked using variance inflation factors (VIFs), which remained below 2.0 for all included variables, indicating low collinearity among predictors.

### Ethical considerations

This study analysed publicly available, de-identified DHS datasets that were collected with ethical approval from national institutional review boards and ICF International. The data of the project was provided by the officials of the DHS program on 6 ^th^ January 2025 after project registration and data requisition.

### Patient-public involvement

The research team had no contact with the study participants, because the data used in this study was retrieved from the DHS data repository. Moreover, participants were not involved in the design, conduct, reporting, or dissemination plans of this study.

## Results

In this study, data of 541,707 children under five years from 62 LMICs (2006–2024) were analysed to measure the prevalence and determinants of CFM and its types.

### Prevalence of paediatric malnutrition at global, regional and national levels

The global prevalence of malnutrition among children under five years between the year 2006 and 2024 was 43.9%.

In general, more than a quarter of children in every region of the world have malnutrition. The highest prevalence of malnutrition was observed in children of SASEA and Oceania region, where more than half of the children exhibit malnutrition. Prevalence of malnutrition in SSA and LAC region was 39.1% and 30.5%. However, CA reported lowest malnutrition prevalence (27.4%) among all regions of the world.

At national level, more than half of the children of Timor-Leste (66.9%), Yemen (59.7%), Burundi (59.6%), Niger (57.5%), India (55.8%), Congo Democratic Republic (51.2%), Chad (51.2%), Guatemala (50.8%), and Papua New Guinea (50.6%) had malnutrition. However, the lowest prevalence of malnutrition was reported in Turkey, i.e., 15.7%. Moreover, Dominican Republic (16.9%), Colombia (19.1%), Jordan (19.6%), Gabon (23.2%), and Gambia (24.3%) also reported malnutrition in less than a quarter of paediatric population.

### Standalone and coexisting forms of paediatric malnutrition at global, regional, and national levels

The global prevalence of CFM was 2.1% lower than SFM (CFM=20.9%~SFM=23%). In general, the SFM among children is more predominant in most of the regions of the world, except SASEA. The prevalence of paediatric CFM reported from SASEA region was 29.6%, which is 6.6% higher than does the SFM (23%). However, children living in CA (8.1%) and LAC region (6.2%) demonstrated lowest prevalence of CFM.

Between CFM and SFM distribution, Bangladesh, Chad, India, Niger, Timor-Leste, and Yemen showed higher CFM prevalence than SFM. Pakistan and Senegal showed almost equal proportion of both CFM and SFM. However, prevalence of CFM in other countries observed was lower than the SFM ( Table 2).

**Table 2.** Global, regional, and national prevalence of malnutrition among neonates, infants, and children.

Country name	Survey year	Total sample size	Malnourished children	Children with SFM ^1^	Children with CFM ^2^	CUW ^3^	CUS ^4^	CUWS ^5^	CSO ^6^
***Global***		*541,707*	*43.91% (42.60-45.19)*	*22.6% (21.7 – 23.5)*	*21.30% (20.41 – 22.22)*	*4.22% (3.80 – 4.61)*	*11.81% (11.13 – 12.47)*	*3.2% (2.85 – 3.55)*	*2.1% (1.82 – 2.38)*
**Central Asia**									
Central Asia		9,860	27.4% (26.37 – 28.43)	19.3% (18.44 – 20.16)	8.1% (7.54 – 8.66)	1.8% (1.54 – 2.06)	3.2% (2.85 – 3.55)	0.7% (0.54 – 0.86)	2.4% (2.1 – 2.7)
Kyrgyz Republic	2012	4,016	27% (25.98-28.02)	18.2% (17.36-19.04)	6.8% (6.29-7.31)	0.9% (0.71-1.09)	2% (1.72-2.28)	0.4% (0.28-0.52)	3.5% (3.13-3.87)
Tajikistan	2017	5,844	27.7% (26.67-28.73)	18.6% (17.75-19.45)	9.1% (8.51-9.69)	2.4% (2.10-2.70)	4% (3.61-4.39)	0.8% (0.62-0.98)	1.7% (1.44-1.96)
**Latin America & Caribbean region**									
Latin America & Caribbean region		64,671	30.5% (29.42 – 31.58)	24.3% (23.33 – 25.27)	6.2% (5.71 – 6.69)	0.4% (0.28 – 0.52)	5.3% (4.85 – 5.75)	0.4% (0.28 – 0.52)	1% (0.8 – 1.2)
Bolivia	2008	7,716	35.1% (33.94-36.26)	28.6% (27.55-29.65)	6.5% (6.00-7.00)	0.4% (0.28-0.52)	3.2% (2.85-3.55)	0.4% (0.28-0.52)	2.5% (2.19-2.81)
Colombia	2010	15,969	19.1% (18.24-19.96)	15.5% (14.73-16.27)	3.6% (3.23-3.97)	0.3% (0.19-0.41)	2.6% (2.28-2.92)	0.2% (0.11-0.29)	0.5% (0.36-0.64)
Dominican Republic	2013	3,187	16.9% (16.09-17.71)	13.3% (12.59-14.01)	3.6% (3.23-3.97)	0.7% (0.54-0.86)	1.8% (1.54-2.06)	0.2% (0.11-0.29)	0.9% (0.71-1.09)
Guatemala	2014-15	11,774	50.8% (49.40-52.20)	37% (35.81-38.19)	13.8% (13.07-14.53)	0.2% (0.11-0.29)	11.7% (11.03-12.37)	0.5% (0.36-0.64)	1.4% (1.17-1.63)
Guyana	2009	1,556	29.4% (28.34-30.46)	18.5% (17.66-19.34)	10.9% (10.25-11.55)	2% (1.72-2.28)	6.1% (5.62-6.58)	1% (0.80-1.20)	1.8% (1.54-2.06)
Haiti	2017	5,583	27.7% (26.67-28.73)	18.3% (17.46-19.14)	9.4% (8.80-10.00)	1% (0.80-1.20)	6.4% (5.90-6.90)	1% (0.80-1.20)	1% (0.80-1.20)
Honduras	2011-12	9,973	29% (27.94-30.06)	22% (21.08-22.92)	7% (6.48-7.52)	0.5% (0.36-0.64)	5.5% (5.04-5.96)	0.4% (0.28-0.52)	0.6% (0.45-0.75)
Peru	2012	9,213	25.9% (24.90-26.90)	22% (21.08-22.92)	3.9% (3.51-4.29)	0.3% (0.19-0.41)	2.8% (2.47-3.13)	0.3% (0.19-0.41)	0.5% (0.36-0.64)
**North Africa, West Asia and Europe**									
North Africa, West Asia and Europe		40,151	41.4% (40.14 – 42.66)	22.1% (21.18 – 23.02)	19.3% (18.44 – 20.16)	3.1% (2.75 – 3.45)	9.5% (8.9 – 10.1)	2.7% (2.38 – 3.02)	4% (3.61 – 4.39)
Albania	2017-18	2,393	25.5% (24.51-26.49)	21.8% (20.88-22.72)	3.7% (3.32-4.08)	0.4% (0.28-0.52)	0.6% (0.45-0.75)	0.2% (0.11-0.29)	2.5% (2.19-2.81)
Armenia	2015-16	1,545	25.8% (24.80-26.80)	20.7% (19.81-21.59)	5.1% (4.66-5.54)	0.6% (0.45-0.75)	1% (0.80-1.20)	0.2% (0.11-0.29)	3.3% (2.94-3.66)
Azerbaijan	2006	1,942	36.5% (35.32-37.68)	20.6% (19.71-21.49)	15.9% (15.12-16.68)	2% (1.72-2.28)	4.4% (3.99-4.81)	0.9% (0.71-1.09)	8.6% (8.03-9.17)
Egypt	2014	13,681	38.9% (37.68-40.12)	26.3% (25.29-27.31)	12.6% (11.90-13.30)	1.8% (1.54-2.06)	2.7% (2.38-3.02)	0.6% (0.45-0.75)	7.5% (6.96-8.04)
Jordan	2017-18	4,927	19.6% (18.73-20.47)	15.6% (14.83-16.37)	4% (3.61-4.39)	0.5% (0.36-0.64)	1.4% (1.17-1.63)	0.3% (0.19-0.41)	1.8% (1.54-2.06)
Turkey	2018	2,039	15.7% (14.92-16.48)	13.9% (13.17-14.63)	1.8% (1.54-2.06)	0.4% (0.28-0.52)	0.7% (0.54-0.86)	0.1% (0.04-0.16)	0.6% (0.45-0.75)
Yemen	2013	13,624	59.7% (58.19-61.21)	22% (21.08-22.92)	37.7% (36.50-38.90)	6.2% (5.71-6.69)	23.4% (22.45-24.35)	6.8% (6.29-7.31)	1.3% (1.08-1.52)
**Oceania**									
Oceania		3,188	50.6% (49.21-51.99)	27.8% (26.77-28.83)	22.8% (21.86-23.74)	4% (3.61-4.39)	11.4% (10.74-12.06)	3% (2.66-3.34)	4.4% (3.99-4.81)
Papua New Guinea	2018	3,188	50.6% (49.21-51.99)	27.8% (26.77-28.83)	22.8% (21.86-23.74)	4% (3.61-4.39)	11.4% (10.74-12.06)	3% (2.66-3.34)	4.4% (3.99-4.81)
**South Asia & Southeast Asia**									
South Asia & Southeast Asia		225,434	52.6% (51.18 – 54.02)	23% (22.1 – 23.9)	29.6% (28.53 – 30.67)	7.3% (6.77 – 7.83)	15.3% (14.53 – 16.07)	5.1% (4.66 – 5.54)	1.9% (1.63 – 2.17)
Bangladesh	2022	4,087	36.5% (35.32-37.68)	16.9% (16.09-17.71	19.6% (18.73-20.47)	4.7% (4.28-5.12)	10.9% (10.25-11.55)	3.5% (3.13-3.87)	0.5% (0.36-0.64)
Cambodia	2022	3,755	33.8% (32.66-34.94)	17.6% (16.78-18.42)	16.2% (15.41-16.99)	4.4% (3.99-4.81)	8% (7.45-8.55)	2.1% (1.82-2.38)	1.7% (1.44-1.96)
India	2019-21	198,802	55.8% (54.34-57.26)	25.3% (24.31-26.29)	30.5% (29.42-31.58)	7.6% (7.06-8.14)	15.6% (14.83-16.37)	5.3% (4.85-5.75)	2% (1.72-2.28)
Maldives	2016-17	2,350	29.6% (28.53-30.67)	17% (16.19-17.81)	12.6% (11.90-13.30)	4.1% (3.70-4.50)	6.1% (5.62-6.58)	1.7% (1.44-1.96)	0.7% (0.54-0.86)
Myanmar	2015-16	4,199	38.1% (36.89-39.31)	20.5% (19.61-21.39)	17.6% (16.78-18.42)	3.2% (2.85-3.55)	12.3% (11.61-12.99)	1.6% (1.35-1.85)	0.5% (0.36-0.64)
Nepal	2022	2,586	34.1% (32.96-35.24)	17.6% (16.78-18.42)	16.5% (15.70-17.30)	2.9% (2.57-3.23)	10.2% (9.57-10.83)	3.2% (2.85-3.55)	0.2% (0.11-0.29)
Pakistan	2017-18	4,098	44.1% (42.80-45.40)	22.1% (21.18-23.02)	22% (21.08-22.92)	2.3% (2.00-2.60)	15.7% (14.92-16.48)	2.8% (2.47-3.13)	1.2% (0.99-1.41)
Timor-Leste	2016	5,557	66.9% (65.30-68.50)	25.1% (24.12-26.08)	41.8% (40.53-43.07)	8.9% (8.32-9.48)	21.4% (20.49-22.31)	8.1% (7.54-8.66)	3.4% (3.04-3.76)
**Sub Saharan Africa**									
Sub Saharan Africa		**198,403**	39.1% (37.87 – 40.33)	22.1% (21.18 – 23.02)	17% (16.19 – 17.81)	2.4% (2.10 – 2.70)	10.9% (10.25 – 11.55)	2.3% (2 – 2.6)	1.4% (1.17 – 1.63)
Angola	2015-16	6,268	44.6% (43.29-45.91)	25.8% (24.80-26.80)	18.8% (17.95-19.65)	1.5% (1.26-1.74)	13.7% (12.97-14.43)	2% (1.72-2.28)	1.6% (1.35-1.85)
Benin	2017-18	11,631	37.8% (36.59-39.01)	22.1% (21.18-23.02)	15.7% (14.92-16.48)	1.9% (1.63-2.17)	11.2% (10.54-11.86)	2% (1.72-2.28)	0.6% (0.45-0.75)
Burkina Faso	2021	5,712	32.8% (31.68-33.92)	16.6% (15.80-17.40)	16.2% (15.41-16.99)	4.7% (4.28-5.12)	7.9% (7.35-8.45)	2.9% (2.57-3.23)	0.7% (0.54-0.86)
Burundi	2016	6,039	59.6% (58.09-61.11)	30.5% (29.42-31.58)	29.1% (28.04-30.16)	1% (0.80-1.20)	23.9% (22.94-24.86)	3.4% (3.04-3.76)	0.8% (0.62-0.98)
Cameroon	2018	4,475	40.2% (38.96-41.44)	25.1% (24.12-26.08)	15.1% (14.34-15.86)	1.6% (1.35-1.85)	7.8% (7.25-8.35)	1.2% (0.99-1.41)	4.5% (4.08-4.92)
Chad	2014-15	9,826	51.2% (49.80-52.60)	22.5% (21.57-23.43)	28.7% (27.65-29.75)	4.2% (3.80-4.60)	18.1% (17.27-18.93)	5.3% (4.85-5.75)	1.1% (0.89-1.31)
Comoros	2012	2,387	45.1% (43.78-46.42)	25.9% (24.90-26.90)	19.2% (18.34-20.06)	3.8% (3.42-4.18)	8.6% (8.03-9.17)	2.2% (1.91-2.49)	4.6% (4.18-5.02)
Congo	2011-12	4,475	31.7% (30.60-32.80)	20.1% (19.22-20.98)	11.6% (10.93-12.27)	1.6% (1.35-1.85)	7.6% (7.06-8.14)	1% (0.80-1.20)	1.4% (1.17-1.63)
Congo Democratic Republic	2012-14	8,059	51.2% (49.80-52.60)	27.2% (26.18-28.22)	24% (23.04-24.96)	2.7% (2.38-3.02)	16.4% (15.61-17.19)	2.4% (2.10-2.70)	2.5% (2.19-2.8)
Cote d'Ivoire	2021	4,734	32.6% (31.48-33.72)	18.5% (17.66-19.34)	14.1% (13.36-14.84)	3.5% (3.13-3.87)	7.2% (6.67-7.73)	2.3% (2.00-2.60)	1.1% (0.89-1.31)
Eswatini	2006-07	2,043	37.7% (36.50-38.90)	29.2% (28.14-30.26)	8.5% (7.93-9.07)	0.7% (0.54-0.86)	3.7% (3.32-4.08)	0.5% (0.36-0.64)	3.6% (3.23-3.97)
Ethiopia	2016	8,768	48.5% (47.14-49.86)	24.8% (23.82-25.78)	23.7% (22.75-24.65)	3.4% (3.04-3.76)	15.9% (15.12-16.68)	3.2% (2.85-3.55)	1.2% (0.99-1.41)
Gabon	2019-21	5,311	23.2% (22.26-24.14)	17.4% (16.58-18.22)	5.8% (5.33-6.27)	1.1% (0.89-1.31)	3% (2.66-3.34)	0.6% (0.45-0.75)	1.1% (0.89-1.31)
Gambia	2019-20	3,805	24.3% (23.33-25.27)	14% (13.27-14.73)	10.3% (9.67-10.93)	2% (1.72-2.28)	6.4% (5.90-6.90)	1.6% (1.35-1.85)	0.3% (0.19-0.41)
Ghana	2022	4,395	25.2% (24.22-26.18)	14% (13.27-14.73)	11.2% (10.54-11.86)	2.5% (2.19-2.81)	6.4% (5.90-6.90)	1.9% (1.63-2.17)	0.4% (0.28-0.52)
Guinea	2018	3,371	39.1% (37.87-40.33)	21% (20.10-21.90)	18.1% (17.27-18.93)	2.8% (2.47-3.13)	10% (9.38-10.62)	1.8% (1.54-2.06)	3.5% (3.13-3.87)
Kenya	2022	17,283	25.2% (24.22-26.18)	15.6% (14.83-16.37)	9.6% (8.99-10.21)	1.9% (1.63-2.17)	5.7% (5.23-6.17)	1.4% (1.17-1.63)	0.6% (0.45-0.75)
Lesotho	2023-24	1,089	40.7% (39.45-41.95)	28.1% (27.06-29.14)	12.6% (11.90-13.30)	0.9% (0.71-1.09)	8.6% (8.03-9.17)	0.7% (0.54-0.86)	2.4% (2.10-2.70)
Liberia	2019-20	2,440	35% (33.84-36.16)	23.5% (22.55-24.45)	11.5% (10.84-12.16)	1.1% (0.89-1.31)	7.2% (6.67-7.73)	1.5% (1.26-1.74)	1.7% (1.44-1.96)
Madagascar	2021	5,756	46.6% (45.26-47.94)	24.3% (23.33-25.27)	22.3% (21.37-23.23)	2.6% (2.28-2.92)	15.7% (14.92-16.48)	3.1% (2.75-3.45)	0.9% (0.71-1.09)
Malawi	2015-16	5,110	42% (40.73-43.27)	29.4% (28.34-30.46)	12.6% (11.90-13.30)	0.8% (0.62-0.98)	8.9% (8.32-9.48)	0.9% (0.71-1.09)	2% (1.72-2.28)
Mali	2018	8,224	35.8% (34.63-36.97)	18.1% (17.27-18.93)	17.7% (16.88-18.52)	3.1% (2.75-3.45)	10.8% (10.16-11.44)	2.9% (2.57-3.23)	0.9% (0.71-1.09)
Mauritania	2019-21	9,795	33.3% (32.17-34.43)	17.9% (17.07-18.73)	15.4% (14.63-16.17)	2.3% (2.00-2.60)	10.8% (10.16-11.44)	1.9% (1.63-2.17)	0.4% (0.28-0.52)
Mozambique	2022-23	3,723	41.8% (40.53-43.07)	26.6% (25.59-27.61)	15.2% (14.44-15.96)	1.1% (0.89-1.31)	11% (10.35-11.65)	1.5% (1.26-1.74)	1.6% (1.35-1.85)
Namibia	2013	1,558	33% (31.87-34.13)	20.1% (19.22-20.98)	12.9% (12.20-13.60)	2.4% (2.10-2.70)	7.6% (7.06-8.14)	1.9% (1.63-2.17)	1% (0.80-1.20)
Niger	2012	4,771	57.5% (56.01-58.99)	21.7% (20.79-22.61)	35.8% (34.63-36.97)	6.6% (6.26-7.10)	20.2% (19.32-21.08)	7.6% (7.06-8.14)	1.4% (1.17-1.63)
Nigeria	2018	11,314	42.7% (41.42-43.98)	21.6% (20.69-22.51)	21.1% (20.20-22.00)	2.1% (1.82-2.38)	15% (14.24-15.76)	3.2% (2.85-3.55)	0.8% (0.62-0.98)
Rwanda	2019-20	3,806	38.4% (37.19-39.61)	29.1% (28.04-30.16)	9.3% (8.70-9.90)	0.5% (0.36-0.64)	6.4% (5.90-6.90)	0.4% (0.28-0.52)	2% (1.72-2.28)
Sao Tome and Principe	2008-09	1,454	45% (43.69-46.31)	28.1% (27.06-29.14)	16.9% (16.09-17.71)	3.5% (3.13-3.87)	6.3% (5.81-6.79)	1.7% (1.44-1.96)	5.4% (4.94-5.86)
Senegal	2023	4,466	28.4% (27.36-29.44	14.2% (13.46-14.94)	14.2% (13.46-14.94)	4.4% (3.99-4.81)	7% (6.48-7.52)	2.5% (2.19-2.81)	0.3% (0.19-0.41)
Sierra Leone	2019	4,100	37.7% (36.50-38.90)	23.7% (22.75-24.65)	14% (13.27-14.73)	1.7% (1.44-1.96)	8.4% (7.83-8.97)	1.9% (1.63-2.17)	2% (1.72-2.28)
South Africa	2016	1,075	37.3% (36.10-38.50)	28% (26.96-29.04)	9.3% (8.70-9.90)	0.9% (0.71-1.09)	3.7% (3.32-4.08)	0.5% (0.36-0.64)	4.2% (3.80-4.60)
Togo	2013-14	3,185	34% (32.86-35.14)	18.7% (17.85-19.55)	15.3% (14.53-16.07)	1.9% (1.63-2.17)	10.2% (9.57-10.83)	2.5% (2.19-2.81)	0.7% (0.54-0.86)
Uganda	2016	4,390	35% (33.84-36.16)	24.3% (23.33-25.27)	10.7% (10.06-11.34)	1.3% (1.08-1.52)	7.1% (6.58-7.62)	1.1% (0.89-1.31)	1.2% (0.99-1.41)
Zambia	2018	8,681	41.7% (40.43-42.97)	28.3% (27.26-29.34)	13.4% (12.68-14.12)	1.3% (1.08-1.52)	8.6% (8.03-9.17)	1% (0.80-1.20)	2.5% (2.19-2.81)
Zimbabwe	2015	4,914	34.2% (33.05-35.35)	24.8% (23.82-25.78)	9.4% (8.80-10.00)	1.4% (1.17-1.63)	5.1% (4.66-5.54)	0.8% (0.80-1.20)	2.1% (1.82-2.38)

Where, 1. SFM = Standalone forms of malnutrition, 2. CFM = Coexisting forms of malnutrition, 3. CUW = Coexistence of underweight with wasting, 4. CUS = Coexistence of underweight with stunting, 5. CUWS = Coexistence of underweight with wasting and stunting, 6. CSO = Coexistence of stunting with overweight/obesity.

**Figure 1.**
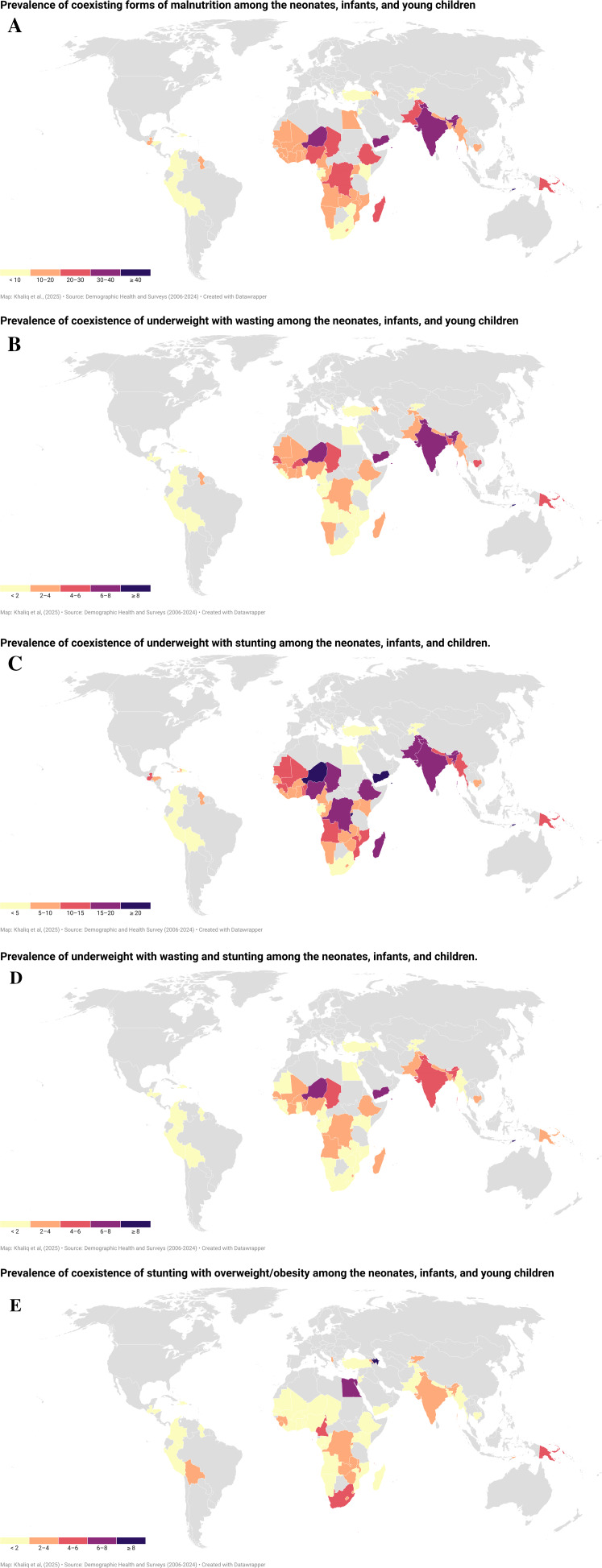
Global, regional, and national prevalence of CFM and its various types among neonates, infants, and children. (A): Global, regional, and national prevalence of CFM among neonates, infants, and children. (B): Global, regional, and national prevalence of CUW among neonates, infants, and children. (C): Global, regional, and national prevalence of CUS among neonates, infants, and children. (D): Global, regional, and national prevalence of CUWS among neonates, infants, and children. (E): Global, regional, and national prevalence of CSO among neonates, infants, and children.

Among 62 LMICs, CFM prevalence was over 10% in 44 countries: Angola, Azerbaijan, Bangladesh, Benin, Burkina Faso, Burundi, Cambodia, Cameroon, Chad, Comoros, Congo, Congo Democratic Republic, Cote d’Ivoire, Egypt, Ethiopia, Gambia, Ghana, Guatemala, Guinea, Guyana, India, Lesotho, Liberia, Madagascar, Malawi, Mali, Maldives, Mauritania, Mozambique, Myanmar, Namibia, Nepal, Niger, Nigeria, Pakistan, Papua New Guinea, Sao Tome & Principe, Senegal, Sierra Leone, Timor-Leste, Togo, Uganda, Yemen, and Zambia. Conversely, Albania, Columbia, Dominican Republic, Jordan, Peru, Turkey reported less than five percent prevalence of paediatric CFM ( Figure 1A).

### Prevalence of various forms of Coexisting Forms of Malnutrition (CFM) at global, regional and national levels

At global level, CUS is the most prevalent type of CFM faced by 11.8% of children, followed by CUW (4.2%), CUWS (3.2%) and CSO (2.1%). Similarly, CUS reported as the most prevalent CFM type at regional level. Highest prevalence of CUS was reported in SASEA (15.3%) followed by Oceania (11.4%) and SSA (10.9%) region, while CA region showed 3.2% CUS cases. With exception to SASEA and SSA, CSO was ranked second most prevalent CFM type in other four regions. The prevalence of CSO ranged from 1% in LAC region to over 4% and 4.4% in NAWAE and Oceania region, respectively. Regarding CUW prevalence, SASEA region reported 7.3% CUW prevalence, but in other five regions, CUW was observed in less than 5% children. However, CUWS was again found highest in SASEA region, while in other regions of the world, specifically CA and LAC region, CUWS affects less than 1% of the population ( Table 2).

Out of 62 LMICs included in this study, the national prevalence of CUW exceeding beyond the global CUW prevalence was observed in nine countries (Bangladesh, Burkina Faso, Cambodia, Chad, India, Niger, Senegal, Timor-Leste, and Yemen). India, Niger, Timor-Leste, and Yemen had 1.5 to 2 folds higher prevalence of CUW among children ( Figure 1B). Similarly, thirteen LMICs (Angola, Burundi, Chad, Congo Democratic Republic, Ethiopia, India, Madagascar, Myanmar, Niger, Nigeria, Pakistan, Timor-Leste, and Yemen) reported CUS prevalence exceeding global CUS estimates. The prevalence of CUS in Burundi, Niger, Timor-Leste, and Yemen was over 20%, which is almost two-folds higher than the global CUS burden ( Figure 1C). However, Bangladesh, Burundi, Burkina Faso, Chad, Ethiopia, India, Nepal, Niger, Nigeria, and Yemen reported higher CUWS estimates than does the global prevalence. Timor-Leste (8.1%) reported highest prevalence of CUWS followed by Niger (7.6%) and Yemen (6.6%) ( Figure 1D). Regarding the prevalence of CSO, seventeen countries, such as Albania, Armenia, Azerbaijan, Bolivia, Cameroon, Comoros, Congo Democratic Republic, Eswatini, Egypt, Guinea, Kyrgyz Republic, Papua New Guinea, South Africa, Sao Tome & Principe, Timor-Leste, Zambia, and Zimbabwe underscored higher CSO prevalence than the global CSO rates. Azerbaijan, Egypt, and Sao Tome & Principe reported over 5% prevalence of CSO among all LMICs ( Figure 1E).

### Association of study covariates with various forms of CFM among neonates, infants, and children

Table 3 reported the association of various covariates with the CUW, CUS, CUWS, and CSO.

**Table 3.** Association of various covariates with the various types of CFM among neonates, infants, and young children.

Variables	CUW ^1^ OR (95% CI)	CUS ^2^ OR (95% CI)	CUWS ^3^ OR (95% CI)	CSO ^4^ OR (95% CI)
Child factors				
Child age				
0 to 11 months	Ref	Ref	Ref	Ref
12 to 23 months	1.47 (1.34-1.61) *	3.00 (2.75-3.27) *	4.01 (3.63-4.43) *	0.21 (0.20-0.23) *
24 to 35 months	1.15 (1.05-1.26) *	3.08 (2.84-3.35) *	2.58 (2.34-2.84) *	0.19 (0.17-0.20) *
36 to 47 months	0.80 (0.73-0.87) *	2.80 (2.58-3.04) *	2.21 (2.01-2.44) *	0.21 (0.19-0.22) *
48 to 59 months	0.75 (0.69-0.82) *	2.10 (1.94-2.27) *	1.57 (1.43-1.73) *	0.23 (0.22-0.25) *
Child sex				
Male	Ref	Ref	Ref	
Female	0.77 (0.73-0.82) *	0.78 (0.74-0.82) *	0.59 (0.56-0.63) *	
Birth order				
Index child	Ref	Ref		Ref
Subsequent child	0.93 (0.87-0.99) *	1.09 (1.03-1.16) *		0.87 (0.83-0.92) *
Birth type				
Singlet		Ref	Ref	
Twins/Triplets		1.39 (1.17-1.65) *	1.81 (1.49-2.20) *	
Birth size				
Small sized				Ref
Average sized				0.87 (0.82-0.93) *
Large sized				0.93 (0.84-1.02)
Diarrhoea				
Yes				Ref
No				0.88 (0.82-0.95) *
Pneumonia				
Yes	Ref			Ref
No	0.88 (0.82-0.96) *			0.91 (0.86-0.96) *
Maternal factors				
Maternal age				
Below 20 years	Ref			
Between 20 to 34 years	1.23 (1.06-1.44) *			
35 years or more	1.22 (1.03-1.45) *			
Maternal education				
No education	Ref	Ref	Ref	Ref
Primary	0.82 (0.75-0.89) *	0.91 (0.83-0.98) *	0.76 (0.69-0.83) *	0.78 (0.73-0.84) *
Secondary	1.00 (0.92-1.08)	0.71 (0.66-0.75) *	0.70 (0.65-0.76) *	1.21 (1.13-1.28) *
Higher	1.07 (0.95-1.21)	0.61 (0.55-0.96) *	0.69 (0.60-0.79) *	1.48 (1.36-1.61) *
Maternal health				
Normal weight		Ref	Ref	Ref
Underweight		0.84 (0.78-0.90) *	1.19 (1.11-1.28) *	0.68 (0.62-0.73) *
Overweight		0.91 (0.83-0.99) *	0.78 (0.71-0.86) *	1.58 (1.49-1.67) *
Obese		0.87 (0.75-1.00)	0.72 (0.60-0.86) *	1.94 (1.80-2.09) *
Household factors				
Wealth index				
Poorest	Ref	Ref	Ref	
Poorer	0.87 (0.80-0.94) *	0.91 (0.84-0.97) *	0.86 (0.79-0.94) *	
Middle	0.86 (0.79-0.94) *	0.83 (0.77-0.90) *	0.82 (0.74-0.89) *	
Richer	0.81 (0.73-0.89) *	0.73 (0.67-0.79) *	0.71 (0.64-0.78) *	
Richest	0.85 (0.76-0.95) *	0.70 (0.63-0.78) *	0.72 (0.64-0.81) *	
Family size				
Small sized family	Ref	Ref	Ref	Ref
Medium sized family	1.06 (1.01-1.11) *	1.09 (1.03-1.16) *	1.09 (1.03-1.17) *	0.85 (0.81-0.89) *
Large sized family	0.86 (0.77-0.95) *	1.11 (1.01-1.22) *	0.96 (0.61-1.18)	0.68 (0.62-0.75) *
Environmental factors				
Water supply				
Improved water supply	Ref	Ref		Ref
Unimproved water supply	0.90 (0.84-0.96) *	1.09 (1.02-1.15) *		0.72 (0.68-0.76) *
Sanitation facility				
Improved sanitation facility	Ref			Ref
Unimproved sanitation facility	0.89 (0.83-0.95) *			0.73 (0.69-0.76) *
Community factors				
Type of place of residence				
Urban				
Rural				

^1^ Where odds of CUW were adjusted with child age, child sex, birth order, pneumonia, maternal age, maternal education, maternal health, wealth index, family size, water supply, and sanitation facility.

^2^ Where odds of CUS were adjusted with child age, child sex, birth order, birth type, maternal education, maternal health, wealth index, family size, and water supply.

^3^ Where odds of CUWS were adjusted with child age, child sex, birth type, maternal education, maternal health, wealth index, and family size.

^4^ Where odds of CSO were adjusted with child age, birth order, birth size, diarrhoea, pneumonia, maternal health, family size, water supply, and sanitation facility.

Overall, the relationship of most covariates with CUW, CUS, and CUWS was almost consistent. An increased odds of CUW, CUS, and CUWS was observed in children aged between 12 to 35 months compared with children aged below 12 months. However, lower odds of CUW, CUS, and CUWS were seen in female children, children of mothers with primary education, and in those with improved SES compared with male children, no education, and poorest SES. Despite various similar associations, differences were still observed.

Compared to index children, subsequent children had lower odds of CUW (0.93; 0.87–0.99) but higher odds of CUS (1.09; 1.03–1.16), with no significant association for CUWS. Twins and triplets consistently faced high risk of CUS (1.39; 1.17–1.65) and CUWS (1.81; 1.49–2.20) but showed no significant association with CUW. Birth size and diarrhoea were not related to any form of CFU, whereas a negative history of pneumonia/respiratory illness reduced the odds of CUW (0.88; 0.82–0.96). Compared to children of teenage mothers, those born to mothers aged 20 years or older were more likely to experience CUW, while maternal age showed no clear effect on CUS or CUWS. However, a complex association between maternal BMI and different CFU types was noticed, i.e., children of overweight/obese mothers had lower odds of CUS and CUWS, but among underweight mothers, the odds remained low for CUS but high for CUWS. Similarly, family size patterns also diverged—medium-sized families were at greater risk across all three forms, but large families showed reduced risk for CUW and CUWS, while presenting the highest risk for CUS.

Compared to children below 12 months, those aged 12–59 months had around 80% lower odds of CSO. Subsequent birth order (13%; 8%–17%) and average birth size (13%; 7%–18%) were also associated with reduced risk. Negative history of diarrhoea and pneumonia/respiratory illness in the past 14 days lowered the odds by 12% (5%–18%) and 9% (4%–15%), respectively. Higher maternal education (secondary and above) and maternal overweight/obesity increased CSO risk, while primary or lower education and underweight mothers were protective to CSO. Moreover, unimproved water and sanitation facilities and medium-to-large family size were also associated to reduce the odds CSO.

## Discussion

This study presented the first analytic reports on global, regional, and national estimations of the existence of CFM among neonates, infants, and children utilizing the nationally representative data of 62 LMICs between 2006 and 2024. Worldwide, 23% of children under five years face SFM, while CFM affects 20.9%. This reflects that approximately one in five children experience CFM, which is associated with a higher mortality risk than SFM, exacerbating health vulnerabilities in children under five. Unfortunately, the concept of CFM received limited attention in global nutrition surveillance. ^31, 32^ Addressing CFM among under five in LMICs will help to achieve WHA 2025 (World Health Assembly) global nutrition targets and 2030 SDG target 2. ^31– 33^

The prevalence of malnutrition varied significantly across different regions of the world. ^2^ Globally, more than half of malnourished children are residents from SASEA and Oceania region ( Table 2). This contrasts with the WHO/JME 2025 findings, which stated SASEA and SSA region as the hub for malnourished children. The contrasting finding of this study was because of unavailability of DHS datasets of countries of Oceania region, except PNG. ^34^ Reliance of only DHS datasets and inclusion of PNG dataset for analysis potentially misrepresent the regional estimates and biasing the inter-regional comparisons. ^35^

The prevalence of CFM across different regions of the world, was either equal to or lower than that of SFM, except SASEA region, where CFM predominated SFM ( Table 2). Worldwide, one out of every third child from SASEA region, and one out of every fifth child from Oceania, SSA, and NAWAE regions is vulnerable to CFM. Khaliq et al. in their review showed high CFM burden in SASEA and SSA region. ^4^ Moreover, the national estimates CFM in most of the countries of SASEA (Timor-Leste, India, Pakistan), SSA (Niger, Burundi, Chad, Congo Democratic Republic, Ethiopia, Madagascar, Nigeria), and Oceania (PNG) region was far beyond the global CFM estimates. However, the CFM prevalence measured in this study is around 4-5-folds higher than the GNR estimates, because in GNR measured CFM burden only among stunted children. ^4, 18, 36, 37^ Moreover, this study also tested WHO hypothesis that an underweight child is at risk of either wasting or stunting or both ^7^ The high burden of CFM in most of the countries of SASEA and SSA regions is exacerbated by political instability, limited healthcare infrastructure, and persistent food insecurity, ^38^ compounded by urban-rural disparities, high rates of female illiteracy and dietary shifts in India. Events like the 2015–2016 El Niño, the 2021 floods in Timor Leste, ^39, 40^ and the Covid-19 pandemic has grown insecurity and posed challenges to tackle malnutrition in all its form. ^37^ However, within the NAWAE region, heterogeneity with stark contrast among countries was observed. Turkey and Yemen both are from NAWAE region, but CFM prevalence in Turkey was 1.8%, but in Yemen, it was 37.7%. This highlights how political stability, conflict, and governance significantly impact nutritional outcomes. ^41^ This heterogeneity in the prevalence of CFM was also observed in SSA and LAC region. Differences in the SES inequalities, ineffective governance, and nutritional deficits all contribute to an increased uneven distribution of CFM within the same region. ^42^ These disparities highlight the need for context-specific interventions. Among all regions of the world, LAC and CA regions had nearly two to four times lower prevalence of CFM than SFM. Thereby, suggesting more targeted nutritional challenges and reflected the region’s progress in reducing poverty and implementing effective nutrition programs over recent decades. ^43^

Among different CFM types, CUS is highly prevalent in all regions. SASEA (15.3%) showed highest burden of CUS, followed by Oceania (11.4%) and SSA (10.9%). The high prevalence of CUS in these regions depicted malnutrition as a chronic and intergenerational issue. Moreover, the JME 2025 report also raised concern of stunting in Africa from 61.7 million to 64.8 million children between 2012 and 2024. ^31, 32^ Apart from CUS, cases of other CFM types were also observed in different regions of the world. Across all regions of the world, SASEA, and SSA regions have relatively more percentage of children with CUW and CUWS. Presence of these forms of CFM exemplifies the severity of both acute (CUW) and chronic (CUS) forms of undernutrition in these regions. ^37, 40^ Children of Timor-Leste (8.1%), Niger (7.6%), Yemen (6.8%), India (5.3%), and Chad (5.3%) are at high risk of CUWS. Previous studies and reports also supported CUWS prevalence exceeding beyond 5% among children of these countries. ^4, 18, 37^ The increased burden of CUWS depicted increased vulnerability to infectious diseases, nutritional deficiency, and mortality among children under five years. ^44– 47^ However, other regions of the world, such as CA, LAC, NAWAE and Oceania showed relatively higher prevalence of CSO than does CUW and CUWS. The relatively high prevalence of CSO in Azerbaijan (8.6%), Egypt (7.5%), Sao Tome and Principe (5.4%), Cameros (4.6%), Cameroon (4.5%), PNG (4.4%), and South Africa (4.2%). This high prevalence of CSO was also observed in our previous study for all countries. ^4, 15, 18^ These findings align with the “nutrition transition theory” proposed by Popkin, which suggests that as countries develop economically, they experience shifts in dietary patterns and physical activity levels that can lead to the simultaneous presence of undernutrition and over nutrition within the same population. ^48, 49^ This burdensome situation complicates the design of programs, clinical management, and resource allocation, which most health systems are not well prepared to confront. In addition, the overlapping CFM with increased risks of communicable diseases exacerbates child health and survival pressure, particularly in already weak settings. ^50^

Children between the age of 12 to 35 months have higher odds of being CUW, CUS, and CUWS ( Table 3). These findings overlap with the dietary transition, increased pathogen exposure, and increased nutritional demands, underscoring “first 1000 days” as a critical window for nutritional intervention. ^36^ The risk of CFM among preschool children reflects the need for nutrition-specific (exclusive breastfeeding advocacy, adherence to complementary feeding, micronutrient supplementation, deworming of intestinal parasites, and nutritional education) and nutrition-sensitive (provision of safe water & sanitation, community education, women’s empowerment, poverty alleviation, and multisectoral collaboration) interventions. ^51^ Between male and female children, the female child had overall lower risks of various forms of CFM as compared to male’s child. Several biological and social factors such as, increase metabolic rates, more protein requirements, and substantially faster growth trajectories than females, potentially making them more vulnerable to nutritional deficiencies especially in low resources settings. ^52, 53^ Moreover, males typically have weaker immune responses that can compromise nutritional status. ^53^

A significant increase in the odds of CUS and CUWS was observed among twins/triplets’ children compared with singleton children, because these children demand increased nutrition during pregnancy and infancy. ^54^ However, the relationship between birth order and CFM was bidirectional. In our analysis subsequent child showed 7 % less likelihood for CUW (acute form) but 9% higher likelihood of CUS (chronic form). Also, there is 13% less chance of developing CSO in subsequent child as evident in our study. A study in Peru found that later-born children had better dietary diversity due to maternal learning. ^55^

No significant association of diarrhoeal disease with any form of CFM was observed. However, different studies reported a bidirectional and synergistic relationship between diarrhoea and malnutrition. ^56, 57^ The lack of association between diarrhoeal disease and CFM might be due to survivorship bias in DHS surveys. Some studies suggest that severely malnourished children with diarrhea/pneumonia may die prematurely, leaving a surviving sample of healthier, less malnourished children in cross-sectional surveys leading to underrepresentation in surveys. ^58– 60^ A study in Bangladesh found that wasted children had nine times higher mortality from diarrhea, meaning many severely malnourished children die before being measured in surveys. ^59^ Surveys like DHS barely investigate the cause of deaths among young children. ^60^ Moreover, families with healthier children may be more likely to report illnesses. ^61^ However, an absence of pneumonia/respiratory illness noticeably reduced the odds of CUW and CSO. Similar evidence was received from a longitudinal study, which demonstrated increased risk of pneumonia among malnourished children. ^62^

Maternal education offers protection to a child against CFM ( Table 3). Maternal education reduces child malnutrition by enhancing nutrition knowledge, such as exclusive breastfeeding and diverse diet practices, effective healthcare access, and economic stability, particularly in rural areas of LMICs. ^63^ However, it has no consistent effect on over nutrition due to dominant environmental factors like urbanization and nutritional transitions by more processed food access, and intergenerational effects. ^49, 64, 65^ Other maternal factors, such as maternal age over 20 years showed higher odds of CUW. A meta-analysis supported that mothers aged over 35 years had 12% higher risk of low birth weight, a precursor to underweight. ^66^ Moreover, maternal obesity, at once end reduced the odds of CUS, and CUWS, but on other end increased the risk of CSO by 58%. This was like the finding of Putri, el al (2025), in which they indicated 94% chances of developing CSO in children of obese mother. ^67^

The global malnutrition rate decreases from 52% in the poorest SES to 32.3% in the richest SES, with marked regional variations. ^29^ An improvement in the SES significantly reduces the odds of CFM in children ( Table 3). This depicts how economic disparities are linked with nutritional inequities. ^68^ However, a JAMA Network Open (2023) study showed weak association between economic growth and child malnutrition in LMICs. ^69^ A complex relationship of CFM with family size was observed. Children from medium sized families have higher odds of various forms of CFU, while the odds of CSO are remarkably reduced in children of both medium and large sized families. This may be due to medium-to-large size families face constraints in food allocation among children, limited parental and health care leading to chronic malnutrition especially in later born children. ^70, 71^

Urban-rural disparities in child malnutrition are consistent across all global regions. Between urban-rural regions, there is a significant difference in the CFM prevalence (23.7% in rural~14.3% in urban). Children of LAC and NAWAE regions showed pronounced urban-rural nutritional disparities among all regions. ^29^ Conversely, in CA region, urban children had higher CFM prevalence than rural children. This exceptional finding suggests different underlying dynamics in this region that could involve rapid urbanization with poor infrastructure, urban poverty and inequality, varied food systems or cultural practices, migration patterns affecting urban populations. ^72^ Environmental factors, particularly access to improved WASH facilities, showed mixed results in this study. This was particularly concerning children from households with unimproved water supply and unimproved sanitation facilities that showed varied results for CUW and CSO. However, the association of unimproved water facilities showed 9% increased risk of developing CUS when improved water facilities were kept as reference ( Table 3). These findings align with pathways of enteric infections and environmental enteric dysfunction. ^73^ This complexity is increasingly recognized in ecological models of child health that consider how environmental factors interact with other social determinants. ^74^

### Study strengths

The key strengths of this study include its large sample size, global coverage, and uniform methodology, which enable meaningful cross-national and cross-time comparisons. The datasets used in this study are well-known for their rigor, because of use of standardized & validated questionnaire, experienced & trained data collectors, and stratified cluster-sampling method. ^27, 35^ Similarly, use of homogenous and standardized measures for data screening, cleaning, coding, categorization, and analysis have improved the validity and reliability of the result. Use of sample-weights adjustments, and regression analysis improved the robustness and accuracy of associations between CFM and covariates.

### Study limitations

Despite of large sample size and inclusion of most of the LMICs, this study findings cannot be generalized because of underrepresentation of Oceania, CA and Southeast Asia region. Some countries (Albania, Armenia, Azerbaijan, Colombia, Dominican Republic, Gabon, Guatemala, Jordan, Namibia, Maldives, Peru, South Africa, and Turkey), which are now not classified as upper-middle-income countries (UMICs) according to World Bank classification were also included in this study, because they either received USAID assistance or were LMICs during survey period or are still considered as LMIC under the broader umbrella of global health. ^75– 77^ The pooled data spans 18 years (2006-2024) limit to represent the current and updated nutrition profile of many countries and regions. Exclusion of around 10-15% children either because of incomplete anthropometry or anthropometric outliers further underrepresent the nutrition profile of the children. Moreover, the sample selected for each country and region, disproportionally presents the paediatric populations, because the DHS targets women of reproductive age rather than children under five for data collection. Furthermore, other important determinants, such as dietary diversity, food insecurity, climate change, and humanitarian crises were not reflected within the scope of this study.

### Policy implications

The escalating burden of CFM among children underscores the need to expand the scope of national and regional nutrition surveys. Incorporating CFM alongside SFM would allow the identification of unidentified and subclinical CFM cases, which need prompt medical and nutritional interventions. Current reliance on *ready-to-use- supplemental-food* (RUSF) and *ready-to-use-therapeutic-food* (RUTF) addresses only acute forms of malnutrition (wasting), leaving gaps for treating other forms of malnutrition, including stunting, underweight, overweight/obesity, and CFM. The policymakers, program managers, clinicians, and other stakeholders should need to prioritize devising nutritional interventions for children with other forms of malnutrition, particularly CFM.

### Recommendation

The CFM in any community, nation and region can be addressed through multisectoral collaboration. Educating communities and empowering women is an essential step to improve dietary practices and reduce CFM, particularly for children aged 12-35 months. Provision of affordable healthcare, alongside improved WASH practices, should be prioritized, with active engagement of community leaders. Food fortification, micronutrient supplementation, and food subsidies for women of reproductive age and children under five would further reduce CFM.

### Future directions

The survey frequency across LMICs is inconsistent, and periodic surveys can better present nutrition profile over time. The DHS implementation bodies should consider underrepresented regions (Oceania, Central Asia, Southeast Asia) for conducting surveys for providing equitable coverage. Integration of survey data with climatic, environmental, and humanitarian factors would provide deeper insight about the external influencers of CFM. Moreover, use of mixed-methods approach, and longitudinal studies will further enhance understanding of context-specific and underlying/unexplored factors associated with CFM in children.

## Conclusion

This study advances the understanding of paediatric malnutrition by emphasizing CFM’s prevalence, determinants, and regional variations, filling a critical gap in global nutrition research. The findings serve as a reminder to reinvest and coordinate efforts to ensure that no child, no matter how precious, suffers from malnutrition during their early years of life. Governments and other stakeholders in the global health sector must invest in a well-established system of nutrition surveillance for addressing CFM, informing targeted interventions to improve child health outcomes in LMICs.

## Data Availability

The analyses presented in this study are based on publicly available data from the Demographic and Health Surveys (DHS) Program, which can be distributed under the **CC BY 4.0 International License** for research purposes. For conducting this research, we used nationally representative DHS datasets from 62 low- and middle-income countries (LMICs), accessible through the DHS Program website ( https://dhsprogram.com/data/available-datasets.cfm). Figshare: Global, regional and national estimates of coexisting forms of malnutrition among the neonates, infants and young children – A secondary data analysis of Demographic & Health Surveys (DHS) from 2006 to 2024. https://doi.org/10.6084/m9.figshare.30598298.v1. ^29^ This project contains the following extended data:
•
**Supplementary file 1:** Determining the sample size of the study from each country dataset, •
**Supplementary file 2:** Nutritional outcomes and their coding, •
**Supplementary file 3:** Distribution of Coexisting forms of malnutrition across the children of different age groups, •
**Supplementary file 4:** Distribution of Coexisting forms of malnutrition among male and female children, •
**Supplementary file 5:** Distribution of Coexisting forms of malnutrition across the children of mothers with different educational levels, •
**Supplementary file 6:** Distribution of Coexisting forms of malnutrition across the children of different socioeconomic groups, •
**Supplementary file 7:** Distribution of Coexisting forms of malnutrition across the children of urban and rural residence, and •
**Supplementary file 8:** Assessing the unadjusted odds of various types of CFM among the neonates, infants, and young children. Data are available under the terms of the Creative Commons Attribution 4.0 International license (CC-BY 4.0).
