# Supplementary material for: Global, regional and national estimates of coexisting forms of malnutrition among the neonates, infants and young children – A secondary data analysis of Demographic & Health Surveys (DHS) from 2006 to 2024
Source: F1000Res. 2025 Dec 19;14:1414. [Version 1] doi: 10.12688/f1000research.172154.1 (PMC12780520; doi:10.12688/f1000research.172154.1)
Supplement: Peer review history

# Reviewer report: Reviewer response for version 1

**DOI:** 10.5256/f1000research.189855.r443315

**Published:** 2026-01-07

**Authors:** Abdul Azeez Hameed

**Affiliations:** 1 Dhamana Veshi Urban Primary Healthcare Centre, Male, Maldives

i have reviewed the article fully and identified that work clearly and accurately presented the current literature, and sufficient details of methods and analysis were provided to allow replication by others.

 also the statistical analysis and its interpretation were  appropriate and conclusion brought were fully supported by given results, thus this is well and accurately written by the authors

Is the work clearly and accurately presented and does it cite the current literature?

Yes

If applicable, is the statistical analysis and its interpretation appropriate?

Yes

Are all the source data underlying the results available to ensure full reproducibility?

Yes

Is the study design appropriate and is the work technically sound?

Yes

Are the conclusions drawn adequately supported by the results?

Yes

Are sufficient details of methods and analysis provided to allow replication by others?

Yes

Reviewer Expertise:

public health, medicine, general practice

I confirm that I have read this submission and believe that I have an appropriate level of expertise to confirm that it is of an acceptable scientific standard.

# Reviewer report: Reviewer response for version 1

**DOI:** 10.5256/f1000research.189855.r443316

**Published:** 2026-01-07

**Authors:** Sumera Saeed Akhtar

**Affiliations:** 1 University of Otago, Dunedin, Otago, New Zealand

Although the concept of coexisting forms of malnutrition (CFM) has been discussed previously, this paper presents the first comprehensive global, regional, and national mapping of CFM using DHS data from 62 LMICs. Overall, well-written and structured. Definitions (e.g., Table 1) are clear. However, I have some concerns and suggestions, as outlined below.

**Abstract: **Add one sentence in the abstract to state why CFM is more important to track than standalone malnutrition. To clearly state that CFM poses higher mortality risks than standalone malnutrition yet remains underreported in global surveillance.”

**Concern:** However, several previous studies (e.g., Ferreira, Varghese, Fongar) have explored overlapping malnutrition, particularly CSO. The claim of complete novelty would be more credible with clearer delineation of what is specifically “new” beyond broader data coverage.

**Suggestion:** Add a short paragraph in the Introduction or Discussion that clearly explains how this study differs from Ferreira (2020), Fongar et al. (2019) and Varghese et al. (2019)

**Concern: **The paper lacks explicit, testable hypotheses. While its aim is descriptive, logistic regression can be used to make causal inferences. Alternatively, if directional hypotheses exist, state them briefly in the introduction.

**Suggestion:** Add a 1–2 sentence clarification in the Methods section that your regression analysis is exploratory (if no prior hypotheses). Either clearly frame directional hypotheses (e.g., “older children are more likely to be CUWS”), or position regressions purely as exploratory.

**Concern:** May be thinking of redundant words in the “Discussion”. Remove paragraphs that repeat numeric findings already shown in tables (e.g., CUWS prevalence in specific countries). Condense country-specific findings into regional summaries.

**Concern:** Pooling datasets across 18 years assumes stationarity in nutrition determinants—an assumption that likely fails given COVID-19, political instability, etc. The exclusion of dietary diversity, food insecurity, and climate variables limits the explanatory power.

**Suggestion:** Acknowledge temporal heterogeneity in results or add a sentence on time pooling in the limitation section, such as Pooling data from 2006–2024 may obscure temporal changes in malnutrition patterns; however, we aimed to use the most recent DHS data available per country.

**Concern:** Relevant literature used for citation, but some citations are overextended, e.g., referring to past research or hypotheses without specificity.

**Suggestion:** Focus on fewer, more directly relevant citations per claim to maintain sharpness and avoid overloading readers.

Is the work clearly and accurately presented and does it cite the current literature?

Yes

If applicable, is the statistical analysis and its interpretation appropriate?

Yes

Are all the source data underlying the results available to ensure full reproducibility?

Yes

Is the study design appropriate and is the work technically sound?

Yes

Are the conclusions drawn adequately supported by the results?

Yes

Are sufficient details of methods and analysis provided to allow replication by others?

Yes

Reviewer Expertise:

N/A

I confirm that I have read this submission and believe that I have an appropriate level of expertise to confirm that it is of an acceptable scientific standard.
